# Integration method of 3D MR spectroscopy into treatment planning system for glioblastoma IMRT dose painting with integrated simultaneous boost

**DOI:** 10.1186/1748-717X-8-1

**Published:** 2013-01-02

**Authors:** Soléakhéna Ken, Laure Vieillevigne, Xavier Franceries, Luc Simon, Caroline Supper, Jean-Albert Lotterie, Thomas Filleron, Vincent Lubrano, Isabelle Berry, Emmanuelle Cassol, Martine Delannes, Pierre Celsis, Elizabeth Moyal Cohen-Jonathan, Anne Laprie

**Affiliations:** 1 Department of Radiation Oncology and Medical Physics, Institut Claudius Regaud, Toulouse, 31052, France; 2 INSERM UMR 825 Imagerie cérébrale et handicaps neurologiques, Toulouse, 31059, France; 3 Centre Hospitalier Universitaire de Rangueil, Université Toulouse III Paul Sabatier, Toulouse, 31059, France; 4 INSERM UMR1037, CRCT, Institut Claudius Regaud, Toulouse, 31052, France

**Keywords:** MR spectroscopy imaging (MRSI), Glioblastoma, Simultaneous integrated boost intensity modulation radiation therapy (SIB-IMRT),Dose painting

## Abstract

**Background:**

To integrate 3D MR spectroscopy imaging (MRSI) in the treatment planning system (TPS) for glioblastoma dose painting to guide simultaneous integrated boost (SIB) in intensity-modulated radiation therapy (IMRT).

**Methods:**

For sixteen glioblastoma patients, we have simulated three types of dosimetry plans, one conventional plan of 60-Gy in 3D conformational radiotherapy (3D-CRT), one 60-Gy plan in IMRT and one 72-Gy plan in SIB-IMRT. All sixteen MRSI metabolic maps were integrated into TPS, using normalization with color-space conversion and threshold-based segmentation. The fusion between the metabolic maps and the planning CT scans were assessed. Dosimetry comparisons were performed between the different plans of 60-Gy 3D-CRT, 60-Gy IMRT and 72-Gy SIB-IMRT, the last plan was targeted on MRSI abnormalities and contrast enhancement (CE).

**Results:**

Fusion assessment was performed for 160 transformations. It resulted in maximum differences <1.00 mm for translation parameters and ≤1.15° for rotation. Dosimetry plans of 72-Gy SIB-IMRT and 60-Gy IMRT showed a significantly decreased maximum dose to the brainstem (44.00 and 44.30 vs. 57.01 Gy) and decreased high dose-volumes to normal brain (19 and 20 vs. 23% and 7 and 7 vs. 12%) compared to 60-Gy 3D-CRT (*p* < 0.05).

**Conclusions:**

Delivering standard doses to conventional target and higher doses to new target volumes characterized by MRSI and CE is now possible and does not increase dose to organs at risk. MRSI and CE abnormalities are now integrated for glioblastoma SIB-IMRT, concomitant with temozolomide, in an ongoing multi-institutional phase-III clinical trial. Our method of MR spectroscopy maps integration to TPS is robust and reliable; integration to neuronavigation systems with this method could also improve glioblastoma resection or guide biopsies.

## Background

Glioblastoma multiforme (GBM) is the most frequent malignant primary brain tumor in adult patients. Prognosis remains poor with a median survival of 14.6 months following treatment with surgery, external beam radiotherapy (RT), and chemotherapy [1]. Although adjuvant RT increases overall survival, whatever the age or Karnofsky/OMS status of the patient, more than 90% of failures occur within the irradiated volumes [2]. This suggests that the dose conventionally delivered is not sufficient. Therefore, there is interest in increasing the dose to specific and more aggressive parts of the tumor while sparing normal tissue, using new technologies such as intensity-modulated radiation therapy (IMRT) [3,4].

As conventional MRI morphological sequences are insufficient to determine the potential target for a dose escalation [5] other types of imaging are needed, such as metabolic imaging [6,7]. The modality of proton magnetic resonance spectroscopy imaging (MRSI) is a relevant tool to define new targets as it can characterize the biochemical, metabolic and pathological changes in brain tissues [8-11] with the analysis of 3D-multi-voxel array within the MRI lesions and the surrounding normal tissue. MRSI data have been correlated with histopathology and can assess the residual disease after surgical resection in high-grade gliomas [12]. In addition, MRSI parameters were also found to be predictive of survival [13,14].

The most common observation in glioblastoma is the peak corresponding to the choline-containing compounds (Cho) which increases with membrane proliferation, thus reflecting tumor presence and aggressiveness [15]. For relative quantification of MR spectroscopic data, the ratio of Cho over *N*-acetyl-aspartate (NAA, a neurotransmitter only found in normal functioning neurons), is used [16]. The volumes corresponding to MRSI abnormalities and contrast enhancement (CE) were found to predict relapse patterns [17,18], in concordance with our results obtained from a prospective trial [19]. MRSI (index of Cho/NAA ≥ 2) could predict the extent of anatomical and metabolic relapse after radio-chemotherapy in patients with glioblastoma [20]. Therefore, these volumes represent potential radioresistant areas on which subvolume boosting [21] or dose painting by contours [22] is possible.

There are two main issues for the integration of MRSI into a RT treatment planning system (TPS). Firstly, MRSI images obtained from MRI scanners are MR spectroscopic maps overlaid on corresponding anatomical MR images. These images do not conform to DICOM standards,they are not compatible (contrarily to conventional MR images) for automatic image fusion with the planning CT scans. Secondly, the escalation in radiation dose from simultaneous integrated boost (SIB) should be carefully evaluated, in particular for organs at risk (OAR).

We performed this study in order to prepare a multi-institutional phase III prospective clinical trial of glioblastoma dose painting guided by MRSI. This trial will compare two RT treatments in concomitance with temozolomide: one delivering 60 Gy on conventional target volume and the other delivering 60 Gy on conventional target volume and a SIB of 72 Gy on a new target volume specific to MRSI.

In this paper, we propose an integration method of metabolic maps into TPS, overcoming the absence of DICOM 3.0 standard for MRSI, to guide the simultaneous integrated boost. We then compare dosimetry plans of standard 60-Gy treatment in 3D conformational radiotherapy (60-Gy 3D-CRT), 60-Gy in IMRT and the treatment with the dose escalation of 72 Gy in SIB-IMRT. The method that we described in this article can be used for future prospective trials integrating MR spectroscopy in radiotherapy planning treatments.

## Methods

### Patients

The pre-RT data were from 16 patients enrolled in a prospective clinical trial on farnesyl-transferase inhibitors (FTI) [19] associated with radiotherapy to treat glioblastoma. The trial was approved by the local ethics committee and patients provided their written informed consent. They received FTI and standard 3D-CRT. We prospectively performed MRSI acquisition before radiotherapy on this homogeneous group at the same session time that the classical MRI sequences.

### Data acquisition

For all 16 GMB patients, MR imaging was performed on a 1.5 T Magnetom Avanto Siemens scanner (Erlangen, Germany). Pre- and post- gadolinium injected (2 mL/kg body weight) T1-weighted (T1–Gd) and Turbo-Spin-Echo T2-weighted (T2) images were acquired for anatomic MR evaluation with voxel resolution of 0.90x0.90x3.00 mm^3^.

3D-chemical-shift imaging (3D-CSI) for MRSI acquisition consisted of three phase-encoded gradients prior to read-out, resulting in a scan time of 8 min. MRSI acquisition consisted of a Spin-Echo-based sequence with the following parameters: TR/TE = 1500 ms/135 ms for lactate detection, and four excitations,FOV was set at 100×100 mm^2^ for a CSI matrix of 16×16, with eight slices of 25.0 mm thickness, resulting in voxel resolution of 6.25×6.25×25.0 mm^3^, i.e. 1 cm^3^. The 3D-CSI box was positioned to cover the majority of abnormalities and normal appearing tissue, while avoiding regions that could corrupt the spectra - such as bone and subcutaneous lipids. Saturation bands were also positioned around the volume of interest (VOI) to suppress signals from excited regions outside the VOI, and to provide good *in vivo* fat suppression.

CT simulation images for RT planning of all 16 GBM patients were acquired in helical mode with voxel resolution of 0.98×0.98×2.50 mm^3^.

### Data processing

The spectroscopic processing protocol consisted of water substraction, low-pass filtering, frequency shift correction, baseline correction, phase correction and curve fitting in the frequency domain. These steps of spectra processing were performed with the Siemens Syngo MR B17 Spectroscopy application (Erlangen, Germany).

### Consistency analysis

After image processing, additional information specific to MRSI abnormalities was embedded in the normalized and segmented anatomic–metabolic images. Accuracy of automated fusion between CT and anatomic MR images is given to be submillimeter and subdegree with Syntegra toolbox (Pinnacle software version 8.0 m, Philips Medical Systems, Milpitas, CA) [23]. We found it relevant to check all 16 patients’ data sets to determine if normalization and threshold-based segmentation could wrongly influence the fusion process between CT scans and anatomic-metabolic images. Reliability and repeatability of the fusion were assessed for each patient’s data with 10 successive co-registration transformations. The result of the fusion process was visually validated. For consistency analysis, the means of standard deviations (SD) and the means of maximum differences between translation and rotation parameters, along the *x* (left–right), *y* (anterior–posterior), and *z* (head–feet) axes were computed.

### Dose-plan comparisons: 60-Gy 3D-C RT, 60-Gy IMRT and 72-Gy SIB-IMRT

For the treatment plans delivering 60 Gy, i.e. 60-Gy 3D-CRT and 60-Gy IMRT, the gross target volume (GTV1) was defined as the anatomical contrast-enhancing tumor visible on the T1–Gd images. The clinical target volume (CTV1), representing the subclinical tumor involvement, was defined as GTV1 + 17.0-mm expansion including the edema visible on the T2-weighted images. The planning target volume (PTV1) was defined as CTV1 + 3.0-mm margin. The dose calculation was performed according to the conventional prescription of 60 Gy delivered in fractions of 2 Gy for the PTV1.

For the 72-Gy SIB-IMRT treatment plan, the GTV2 was defined as the MRSI abnormalities (Cho/NAA ≥ 2.00). The CTV2 was defined as the GTV2 + 7.0-mm expansion including the contrast-enhancing tumor visible on the T1–Gd images. The PTV2 was defined as the CTV2 + 3.0-mm margin. The dose prescription was the following: 60 Gy on the PTV1 as defined above and 72 Gy on the PTV2 (SIB) delivered in fractions of 2.4 Gy.

We wanted to use the radiobiological advantages of an integrated boost,and therefore, taking into account the alpha/beta = 3 as calculated with the LQ model and the dose equivalent for tumor repopulation, 80 Gy as the 2 Gy per day are equivalent to 30 fractions of 2.4 Gy [24].

The TPS used in this study was Pinnacle version 8.0 m (Philips Medical Systems, Milpitas, CA). The dose was calculated with the collapsed cone convolution-superposition model. For SIB-IMRT, we used the Direct Machine Parameter Optimization module, which directly optimizes the number of monitor units and the multileaf collimator leaves.

For comparison with the treatment plans delivering 60 Gy (60-Gy 3D-CRT and 60-Gy IMRT), six different beam configurations of 72-Gy SIB-IMRT, for all 16 patients’ data sets, were tested: 96 dose-plans were then simulated. The six different beam configurations consisted of the following: configuration A = 3 coplanar beams, configuration B = 3 coplanar beams with different angles from configuration A, configuration C = 5 coplanar beams, configuration D = 7 coplanar beams, configuration E = 9 coplanar beams and configuration F = 5 non-coplanar beams (3 coplanar and 2 non-coplanar beams).

The treatment plans delivering 60 Gy (60-Gy 3D-CRT and 60-Gy IMRT) and the 72-Gy SIB-IMRT plans were compared using the following criteria:

Target coverage (*I*_*1*_), conformity index (*I*_*2*_), and conformation number (*CN*) for quantifying the degree of conformity [25]:

$$\begin{array}{c} I_{1}={PTV}_{95\%}/V_{TOT\_PTV}\\ I_{2}={PTV}_{95\%}/V_{ISO\_95}\\ \;{PTV}_{95\%}=\text{Target volume (PTV) receiving 95\% of the prescription dose}\\ \;V_{TOT\_PTV}=\text{Total volume of target (PTV)}\\ \;V_{ISO\_95}=\text{Isodose volume enclosed by 95\% of the prescription dose}\\ CN=I_{1}\times I_{2} \end{array}$$

Doses received by OAR: maximum dose at 1% of the optic chiasm and brainstem.

The dose-volumes of interest for the normal brain were 18, 36, and 50 Gy given relative to the volume of normal brain (V18, V36 and V50).

### Statistical analysis

Continuous variables were described as their medians and range. A Wilcoxon test compared the paired data. All *p*-values were two-sided, and for all statistical tests, differences were considered significant at the 5% level. Stata (StataCorp LP, Texas, USA) was used for all statistical analyses.

## Results

### MRSI integration into TPS

**Figure 1:**
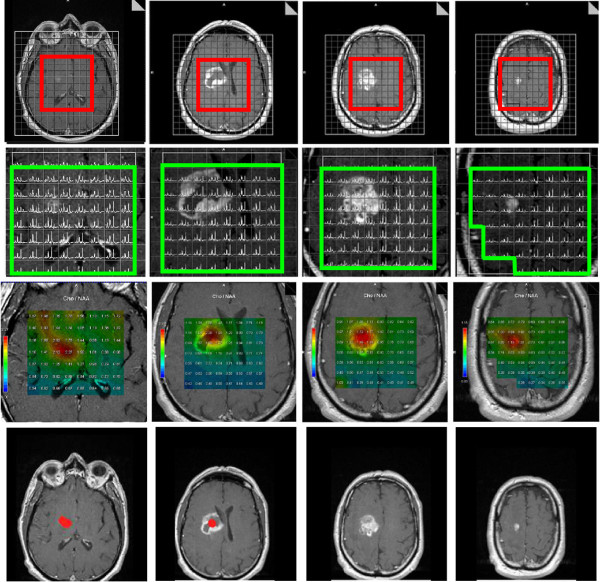
**3D-MRSI acquisition before radiation therapy treatment of a 53 year-old unresected patient with confirmed glioblastoma located in the right capsulo-thalamic region (first row, the volume of acquisition is framed in red).** On the T1-Gd anatomic MR images showing contrast-enhancing disease, the MRSI volume of interest is defined on a voxel by voxel basis,when alteration of metabolites spectra is observed, the voxel is rejected (green frame on second row). The anatomic-metabolic maps are computed from the above defined volume of interest (third row), the maximum Cho/NAA ratio values are encoded in red color and are respectively from left to right 2.27, 2.30, 1.52 and 1.15. The first two metabolite maps which present ratios of Cho/NAA ≥ 2.00 suggest metabolic tumor activity. Regions of interest corresponding to ratio of Cho/NAA ≥ 2.00 are obtained after normalization and threshold based segmentation from the anatomic-metabolic images, these ROIs are highlighted in red (last row). Note on the first image (last row) that the location of the abnormal spectroscopic region is different and below the contrast-enhancing area.

MRSI acquisition from all patients resulted in 3652 voxels (mean = 228.3, SD = 55.6), The Cho/NAA ratio was thoroughly reviewed on a voxel-by-voxel basis: 3120 voxels were considered for the computation of metabolite Cho/NAA maps. Both MR images (Figure 1, first row) and 3D-MRSI were acquired in the same plane in order for co-registration to occur, and the resulting snapshots consisted of anatomic–metabolic images coded under the red–green–blue (RGB) color-space with a DICOM extension format file, but these were not compatible for integration into TPS (Figure 1, third row).

These anatomic–metabolic images were processed with scripts written in Matlab® (MathWorks, Inc., Natick, USA) for integration into TPS. For the image-processing normalization step, the separation of anatomical MR images from the color metabolite ratio maps was made. Conversion of the metabolite ratio maps from RGB to hue-saturation-value (HSV) color-space [26] was performed to retrieve a single quantitative value (hue) from the metabolic map, which was proportional to Cho/NAA. The Siemens Syngo MR B17 Spectroscopy application (Erlangen, Germany) provided the local maximum of Cho/NAA for each CSI slice (i.e. the red color on the corresponding color table), these values were used as inputs to compute global normalization across the entire 3D-MRSI volume of acquisition. The same principle of color-space conversion is possible for other color tables from different MR spectroscopy post-treatment softwares.

To extract the information specific to abnormal MRSI regions, the previously normalized maps were segmented with a threshold value of Cho/NAA ≥ 2.00 [20]. Such segmented regions of interest, with abnormal metabolite index ratios, were then re-mapped onto respective anatomical MR images (Figure 1, last row) with smooth linear interpolation to the final resolution of anatomical MR images. In this study, the 3D-MRSI volume represented 24.84% and 13.63% of the T1–Gd and hyper-T2 volumes, respectively (range: 2.22–54.85%). The integration was finalized with a copy of the DICOM headers from the T1–Gd images into the headers of the anatomic–metabolic images. These normalized and segmented anatomic–metabolic images were generated for all patients, and were successfully imported for fusion with planning CT scans under different TPS and evaluation software for RT (Pinnacle v8.0 m, Eclipse v8.9 and Artiview v2.6).

**Figure 2:**
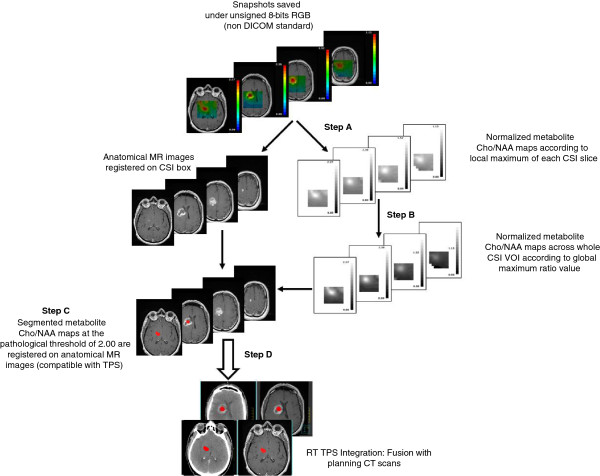
Flow chart of the image processing steps to integrate MRSI-defined regions with abnormal Cho/NAA ratio values into RT TPS.

To summarize, the flow chart of image-processing steps that integrated MRSI-defined regions with abnormal Cho/NAA ratio values into RT TPS is detailed in Figure 2.

### Consistency analysis of MRSI integration into TPS

Results for the reliability and repeatability of the image fusion (10 successive co-registration transformations on each patient’s data) were the following: means SD were found to be 0.19 mm, 0.25 mm, 0.39 mm, 0.25°, 0.37°, and 0.29° and means of maximum differences were found to be 0.59 mm, 0.49 mm, 0.83 mm, 0.82°, 1.15°, and 0.94°,respectively for each translation and each rotation parameter, along the *x* (left–right), *y* (anterior–posterior), and *z* (head–feet) axes.

### Dose-plan comparisons between 60-Gy 3D-CRT, 60-Gy IMRT and 72-Gy SIB-IMRT

**Figure 3:**
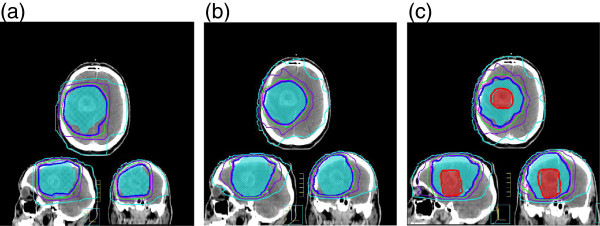
**Comparison of dose plans between 60-Gy 3D-CRT, 60-Gy IMRT and 72-Gy SIB-IMRT. 60-Gy 3D-CRT and 60-Gy IMRT plans (respectively Figures**3a and 3b) have one PTV1 color-washed in blue. The integration of Cho/NAA abnormal volumes defines new target relative to MRSI, i.e. PTV2 color-washed in red (Figure 3c), PTV1 is the same. The isodoses of 68.4 Gy (thick red isodose) and 57 Gy (thick dark blue isodose) represent 95% of the prescribed dose respectively 72 Gy and 60 Gy on the PVT2 and PTV1. The isodose volumes of 54 Gy (pink), 50 Gy (green), 36 Gy (purple) and 18 Gy (light blue) for organs at risk sparing are also plotted.

96 SIB-IMRT treatment plans were simulated and compared with 16 plans of 60-Gy IMRT and 16 standard 3D-CRT plans (Figure 3). Median volumes of PTV1 and PTV2 were respectively 307.76 cm^3^ (range: 84.52–586.96 cm^3^) and 97.63 cm^3^ (range: 34.32–231.17 cm^3^).

Considering the PTV2 of the SIB-IMRT treatment plans, *I*_*1*_, *I*_*2*_, and *CN* were evaluated and configuration C was not statistically different from configurations A, D, E, and F, but higher *I*_*1*_, *I*_*2*_, and *CN* were found compared with the configuration B (respectively, 0.97 vs 0.95, *p* = 0.005,0.79 vs 0.74, *p* = 0.030 and 0.75 vs 0.68, *p* = 0.034). Configuration C was then chosen for dose-plan comparison with standard 60-Gy 3D-CRT and 60-Gy IMRT with 5 beam configuration.

Considering the PTV1, the configuration C shows no significant difference with configurations A, D, E and F but higher *I*_*1*_, *I*_*2*_ and *CN* are found compared with the configuration B (respectively, 0.97 vs 0.95, *p* = 0.005,0.88 vs 0.84, *p* = 0.013 and 0.85 vs 0.82, *p* = 0.001). *I*_*1*_, *I*_*2*_ and *CN* for PTV1 are compared between 60-Gy 3D-CRT, 60-Gy IMRT and 72-Gy SIB-IMRT (configuration C). There is no significant difference for *I*_*1*_ (0.98, 0.95 vs 0.97, *p* > 0.255) but 60-Gy IMRT and 72-Gy SIB-IMRT plans performed significantly better for *I*_*2*_ and *CN* (respectively, 0.91, 0.88 vs 0.75, *p* < 0.010 and 0.84, 0.85 vs 0.72, *p* < 0.035).

**Figure 4:**
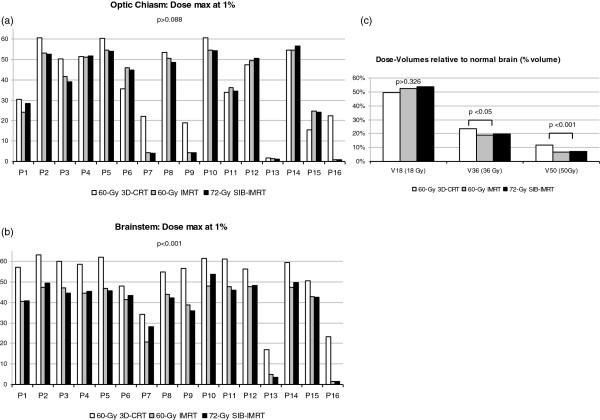
**Comparison of the doses received by OAR between 60-Gy 3D-CRT, 60-Gy IMRT and 72-Gy SIB-IMRT for all patients (P1 to P16).** Doses relative to 60-Gy 3D-CRT are drawn in white, grey for 60-Gy IMRT and black for 72-Gy SIB-IMRT. No significant difference is found when considering the maximum dose received by 1% of the optic chiasm (41.63 vs 45.47 and 42.08 Gy, *p >* 0.088) (Figure 4a). For the brainstem, the maximum dose received by 1% of the organ is significantly lower (57.01 vs 44.30 and 44.00 Gy, *p* < 0.001) in 60-Gy IMRT and 72-Gy SIB-IMRT (Figure 4b). Histograms of dose-volumes relative to normal brain comparing 60-Gy 3D-CRT (white), 60-Gy IMRT (grey) and 72-Gy SIB-IMRT (balck) are shown on Figure 4c. No significant difference is found when considering the V18 (*p* > 0.326). 60-Gy IMRT and 72-Gy SIB-IMRT were significantly smaller for V36 and V50 (19 and 20 vs. 23%, *p* = 0.049,7 and 7 vs. 12%, *p* < 0.001). No significant differences for V36 and V50 were found between 72-Gy SIB-IMRT and 60-Gy IMRT (*p* = 0.605).

When comparing the maximum dose received by OAR, there was no statistically significant difference between 60-Gy 3D-CRT, 60-Gy IMRT and 72-Gy SIB-IMRT for the optic chiasm (*p* > 0.088, Figure 4a). Compared to 60 Gy 3D-CRT, 60-Gy IMRT and 72-Gy SIB-IMRT significantly lowered the dose to the brainstem (*p* < 0.001, Figure 4b).

For doses relative to the normal brain (Figure 4c), the dose-volume V18 was not significantly different, but V36 and V50 were significantly lower with 60-Gy IMRT and 72- Gy SIB-IMRT (*p* < 0.05 and *p* < 0.001).

## Discussion

In this paper, we described an integration method of MRSI for radiotherapy treatment planning in order to perform a clinical trial for glioblastoma dose painting.

At a time when delivering a higher dose to such radioresistant tumors is possible with IMRT, defining the optimal target is of paramount importance.

Several studies with additional boost showed good tolerance for dose escalation [27] and sometimes improved tumor control [3,28-30] but they were not systematically in concomitance with temozolomide and did not perform selective simultaneous integrated boost according to functional or metabolic imaging modalities; to our knowledge the only comparable approach was published by Piroth et al. [27], who described a prospective phase II study that defined the dose escalation (total dose of 72 Gy) with an integrated boost on active tumor as characterized by positron emission tomography (PET) using O-(2-^18^ F]fluoroethyl)-L-tyrosine (FET), for which the results of survival data were comparable to standard treatment [1].

Despite the information contained in MRSI for predicting the site of relapse after radiotherapy [17,20], 3D-MRSI still remains a challenging modality to integrate into TPS. Several attempts have been performed in the last decade [31,32], also in the radiosurgery field [33], but the innovative aspects of the MR spectroscopy integration presented here are the following: a) it is a method for three-dimensional MR spectroscopy that provides global normalization and threshold-based segmentation of the whole 3D-CSI volume of interest,b) it integrates metabolite ratio maps into TPS using co-registration with anatomic MR images, and c) it results in MR spectroscopic pre-defined regions ready to be contoured.

In this study, a consistency analysis was performed to assess the impact of combining MR anatomic and metabolic information on fusion with CT scans, as both MR and MRSI modalities were gathered in the same set of images.

As reliable integration of valuable biological target volumes specific to MRSI into TPS was reached, a dosimetry study was performed to evaluate the impact on the OAR of dose escalation. SIB-IMRT allowed us to perform dose painting by contours for optimal irradiation of target volumes and optimal sparing of OAR [34]. Protection of normal brain tissue is of particular importance for tolerance to dose increases and to prevent radiation necrosis and neurocognitive deficits, as these are significantly correlated with the dose received by the normal brain [35]. Thus, a difficult compromise between radiation necrosis, neurocognitive impairment and tumor control has to be achieved. Although the dose is increased on the target volume, the dose received by OAR is either equivalent (optical chiasm) or significantly lowered with the MRSI guided 72-Gy SIB-IMRT compared to 60-Gy 3D-CRT (brainstem and normal brain) thanks to the IMRT technique. The repercussions of normal brain irradiation for patients with GBM will be more of an issue if patient survival is extended by dose escalation to regions that have a high risk of relapse.

## Conclusions

We describe a reliable method to integrate 3D-MRSI for dose escalation on regions of high-risk of relapse while optimizing OAR sparing. This work represents a novel approach to the treatment of glioblastoma and is the basis of a multi-institutional phase-III prospective clinical trial, which is currently underway to compare conventional treatment delivering 60-Gy versus 72-Gy SIB-IMRT guided by MRSI. This method could also be the basis of other innovative trials integrating MRSI in radiotherapy treatment planning but also in neuronavigation system to improve the GBM resection or guide biopsies.

## Competing interests

The authors declare that they have no competing interests.

## Authors’ contributions

AL, EMCJ and SK participated in the design and coordination of the study. LV, CS and LS participated in the dose-plan comparison analysis. TF contributed to the statistical analysis. LV, CS, LS, AL and SK contributed to the interpretation of the data. SK and AL drafted the article. EMCJ, XF, JAL, VL, IB, EC, MD and PC critically reviewed/revised the article. All authors read and approved the final manuscript.
